# Removal and Recovery of Methyl Tertiary Butyl Ether (MTBE) from Water Using Carbon Nanotube and Graphene Oxide Immobilized Membranes

**DOI:** 10.3390/nano10030578

**Published:** 2020-03-22

**Authors:** Worawit Intrchom, Sagar Roy, Somenath Mitra

**Affiliations:** Department of Chemistry and Environmental Science, New Jersey Institute of Technology, Newark, NJ 07102, USA; wi6@njit.edu (W.I.); sagar@njit.edu (S.R.)

**Keywords:** MTBE separation, functionalized carbon nanotube immobilized membranes (CNIM-f), graphene oxide immobilized membranes (GOIM), sweep gas membrane distillation (SGMD), flux enhancement

## Abstract

Methyl tert-butyl ether (MTBE) is a widely used gasoline additive that has high water solubility, and is difficult to separate from contaminated ground and surface waters. We present the development in functionalized carbon nanotube-immobilized membranes (CNIM-f) and graphene oxide-immobilized membranes (GOIM) for enhanced separation of MTBE via sweep gas membrane distillation (SGMD). Both types of modified membranes demonstrated high performance in MTBE removal from its aqueous mixture. Among the membranes studied, CNIM-f provided the best performance in terms of flux, removal efficiency, mass transfer coefficients and overall selectivity. The immobilization f-CNTs and GO altered the surface characteristics of the membrane and enhanced partition coefficients, and thus assisted MTBE transport across the membrane. The MTBE flux reached as high as 1.4 kg/m^2^ h with f-CNTs, which was 22% higher than that of the unmodified PTFE membrane. The maximum MTBE removal using CNIM-f reached 56% at 0.5 wt % of the MTBE in water, and at a temperature of 30 °C. With selectivity as high as 60, MTBE recovery from contaminated water is very viable using these nanocarbon-immobilized membranes.

## 1. Introduction 

Over 20 million tons of methyl tert-butyl ether (MTBE) is produced and used as a fuel additive around the world every year, and it has contaminated ground and surface water all across the globe [1,2]. Its impact on the environment and human health has been a great concern in the USA and other countries as well [3,4,5,6,7,8,9]. MTBE is highly water soluble, has a low Henry’s Law constant and low sorption constants [2]. These properties also enhance the mobility of MTBE, and make it difficult to separate from water. Methods such as air stripping, adsorption, oxidation processes and pervaporation have been used for MTBE removal [10,11,12]. Air stripping is the conventional method for separating MTBE from water. However, this approach works well only at high temperature, which is energy extensive, and lowering the temperature of MTBE-contaminated feed water can reduce the efficiency of this technology significantly. Additionally, the technology is often not practical if the air stream has to be treated [13]. Although the adsorption of low concentration MTBE on activated carbon can be a challenge, this approach also requires a desorption step [14,15]. Advanced oxidation processes have also been tested, but they can lead to the formation of toxic by-products. Membrane-based processes can overcome some of the shortcomings of the conventional processes mentioned above, and are gaining interest [10]. Membrane-based pervaporation has been applied for MTBE separation from water [16,17]. The results show a high separation factor for MTBE [17], however, the process has relatively low permeate flux [18].

Membrane distillation (MD) is a thermally-driven, membrane-based process, which has been utilized for desalination and wastewater treatment, including volatile compound separation [19,20,21]. The main advantages of MD include high rejection of non-volatile species and relatively low membrane fouling. Furthermore, a low-grade energy, such as solar/geothermal energy, and industrial waste heat, have been efficiently used as a heat source, since MD can be operated at low temperature (30–70 °C) [20,22,23]. A key element of the MD system is the membrane, which directly influences the separation performances. This MD process exploits the differential partial vapor pressure between feed and permeate side as the driving force to transport the vapor molecules through the membrane, where the liquid/membrane interface needs to be hydrophobic to prevent membrane pore blocking and wetting. Understanding these interactions at the membrane interface is important for tailoring the membrane properties [24]. A variety of modification methods have been used to promote the hydrophobicity of the membrane surface [25]. For example, a hydrophobic styrene–butadiene rubber was chemically-coated polyamide [26], while tetrafluoromethane was employed to modify the surface of polyethersulfone membranes by vacuum plasma modification [27]. The hydrophobic materials, such as polypropylene (PP), polyvinylidenefluroride (PVDF), polyethylene (PE) and polytetrafluoroethylene (PTFE) have been used to synthesize MD membranes [28]. However, they tend to be very hydrophobic, and show little affinity for MTBE. We have reported a membrane modification approach using carbon nanotubes (CNTs), f-CNTs and GO [29,30,31,32,33] to improve the characteristics of the membrane, and have shown an excellent adsorption and transport of solutes, leading enhanced flux and selectivity in desalination, as well as the separation of organic solvents from aqueous mixtures [21,23,31]. Although several techniques have been tested for MTBE removal from aqueous solution, few studies have investigated the removal using vacuum MD [34,35]. MTBE separation using SGMD with a nanocarbon-modified membrane at low temperature has much potential for performance improvement, and has not been studied. The objective of this project was to study the removal of MTBE from water via SGMD, using carbon nanotube and a graphene oxide-immobilized membrane.

## 2. Materials and Methods

### 2.1. Chemicals and Materials

Methyl tert-butyl ether (MTBE) (99.8%) was purchased from Sigma Aldrich (Sigma-Aldrich Inc, St. Louis, MO, USA). Flat sheet polytetrafluoroethylene (PTFE) membrane with a polypropylene (PP) supporting layer (0.2 µm pore size, 119 µm thickness, and 74% porosity) was obtained from Adventec Toyo Kaisha, Ltd., (Tokyo, Japan). Multi-walled carbon nanotubes (MWCNTs) (OD 20–30 nm, length 10–30 µm, purity > 95%) were purchased from Cheap Tubes Inc., (Brattleboro, VT, USA). Graphene oxide (GO) was obtained from Graphenea Inc., (Cambridge, MA, USA). Deionized water was used in all experiments.

### 2.2. CNIM-f and GOIM Fabrication and Characterization

The functionalized CNTs (f-CNTs) were prepared by bonding the carboxyl functional group on the MWCNT sidewall through microwave-induced reaction in a Microwave Accelerated Reaction System (CEM Mars), as stated in our previous paper [36].

CNIM-f was fabricated by the procedure, as explained in our recent paper [23]. 1.5 mg of f-CNTs was mixed with 8 g of acetone and uniformly dispersed by an ultrasonic bath for 4 h. The PVDF solution used as a binder was separately prepared by dissolving 0.2 mg of PVDF in 2 g of acetone and added to f-CNTs solution. Immobilization of f-CNTs on the membrane was performed by drop casting of the f-CNTs-PVDF mixture on the membrane surface. The modified membrane was dried under the fume hood overnight. GOIM was also fabricated with the same procedure, except sonication for 10 h.

The membrane morphology and the deposition of CNTs and GO on the membranes were characterized using the scanning electron microscope (SEM, model JSM-7900F, JEOL USA Inc, Peabody, MA, USA). The plain membrane, CNIM-f and GOIM were further characterized by Raman spectroscopy (DXR Raman microscope, Thermo Fisher Scientific, Waltham, MA, USA). The instrument was operated by using 532 nm diode-pumped, solid state (DPSS) laser with laser power of 8 mW, and five scans, with each for 2 s, were performed. The thermal stability of the membranes was examined via thermal gravimetric analysis (TGA, model TGA 8000, PerkinElmer Inc, Hopkinton, MA, USA) using N2 in the temperature range of 30–620 °C, with a heating rate of 10 °C/min. Contact angle measurement used to illustrate the hydrophobic/hydrophilic property of the membrane surfaces was obtained by the water drop method. Water and 2.5% MTBE solution were used in the contact angle measurement. A micro syringe (Hamilton 0−100 μL) was employed to create 2 μL droplets for the measurement as the method described in our previous paper [23]. Surface free energy of the unmodified and modified membranes was calculated from contact angle data following the Owens−Wendt (extended Fowkes) model [37] by using water, ethylene glycol, glycerol and propanol as test liquids [38].

The liquid entry pressure (LEP) of PTFE and modified membranes was measured by a procedure mentioned in the article [39]. The membrane module was connected to a stainless steel chamber (Alloy Products Corp, 185 Psi Mawp) filled with the MTBE solution, by the high-pressure tubing (Masterflex Transfer Tubing, PTFE, 1/8” ID × 1/4” OD). Compressed N2 gas was used to build up the pressure in the chamber, and the increased pressure forced the liquid going into the tube. The pressure that drives the liquid, first entering through the membrane pores, is LEP. The triple measurement was carried out to ensure reproducibility.

### 2.3. Experimental Setup

**Figure 1:**
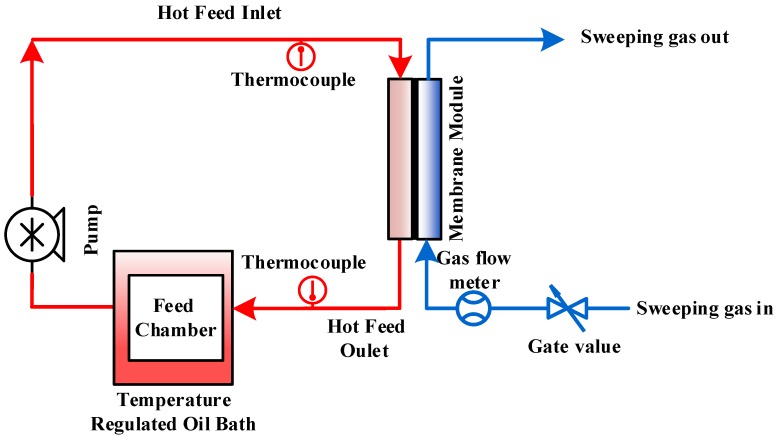
Schematic diagram of the experimental setup for the sweep gas membrane distillation (SGMD).

The sweep gas membrane distillation (SGMD) configuration was employed in this study. The MTBE solution was recirculated around the feed side of the membrane, while a dried-inert gas swept the vapor on the permeate side of the membrane. Before being passed through the permeate side, the sweep air was treated according to the procedure in our previous paper [21]. The SGMD setup in this experiment is shown as the schematic diagram in Figure 1. The MD module had an active contact area of 11.94 cm^2^. An enclosed feed chamber was used to prevent sample loss via evaporation. A peristaltic pump (Cole-Parmer, Masterflex L/S compact pump model 77240-00) was used to recirculate the feed solution. The constant temperature water bath (NESLAB Instruments, Inc., Water Bath Model GP 200) was used to control the temperature of the feed. The temperature sensors (Cole-Parmer, Four-channel data logger thermometer) were employed to continuously monitor the inlet and outlet temperatures of the feed side.

### 2.4. Experimental Procedure

MTBE feed solutions with different concentrations (*w*/*w*) were prepared by adding weighed amounts of MTBE to deionized water. The performances of PTFE and modified membranes were measured under different conditions, i.e., concentrations (0.5, 1.5 and 2.5 wt % MTBE), temperatures (21, 30 and 40 °C), and feed flow rates (20, 30 and 40 mL/min). The air flow rate on the permeate side was kept constant at 2 L/min for all experiments. The change in the feed amount was measured by subtracting the initial and final weight. The concentrations of the MTBE solution were analyzed by a UV Spectrophotometer (UV-1800 UV-Vis Spectrophotometer, Shimadzu Inc, Canby, OR, USA) with λ_max_ at 194 nm. The concentration and weight of the feed solutions before and after the experiments were used to calculate the flux and the selectivity of the membranes. Each experiment was carried out in triplicate to confirm the reproducibility.

## 3. Results and Discussion

**Figure 2:**
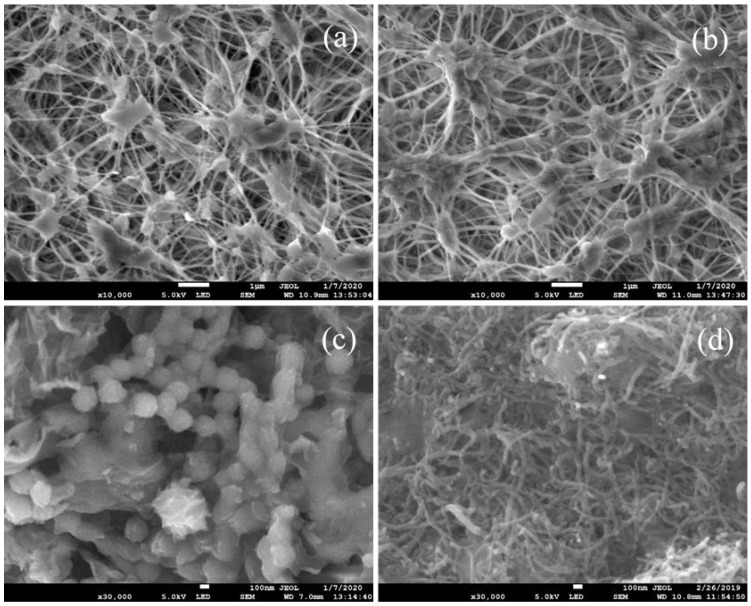
Scanning electron microscopy (SEM) images of (**a**) the feed side of original PTFE membrane, (**b**) the permeate side of original PTFE membrane, (**c**) graphene oxide-immobilized membranes (GOIM) and (**d**) functionalized carbon nanotube-immobilized membranes (CNIM-f).

**Figure 3:**
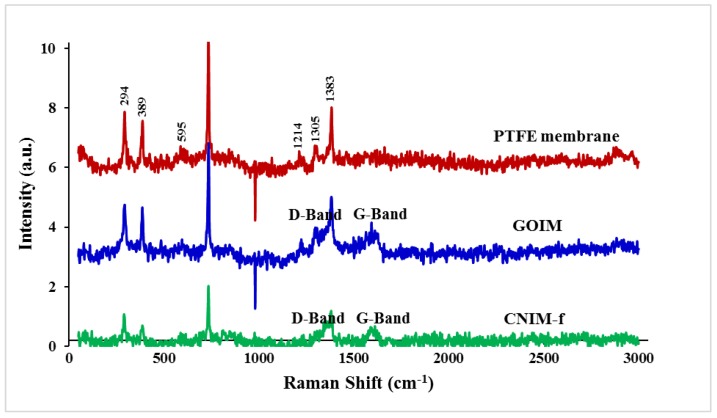
Raman spectra of PTFE membrane, GOIM and CNIM-f.

The SEM images of the plain PTFE membrane, GOIM and CNIM-f are illustrated in Figure 2. Figure 2a,b show the biaxially stretched microporous structure of the feed and the permeate side of the original PTFE membrane, while Figure 2c,d present the morphology of the feed side of the membranes after modification by GO and f-CNTs, respectively. Uniform deposition of GO and f-CNTs was found over the entire membrane surface. Figure 3 presents the Raman spectra of PTFE membrane, GOIM and CNIM-f. The Raman spectrum of the PTFE membrane showed the dominant peaks at 294, 389, 735 and 1383 cm^−1^ [40]. These peaks can also be seen in the spectrum of GOIM and CNIM-f, together with the signature bands of graphitic materials at ~1350 cm^−1^ and ~1595 cm^−1^, called the D and G-band, respectively [41].

**Figure 4:**
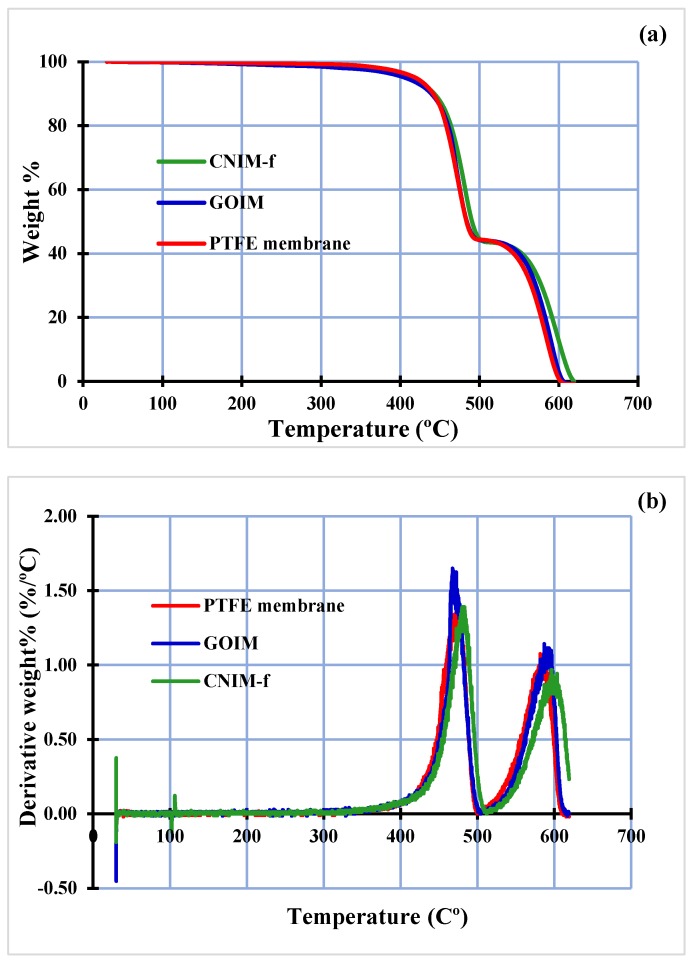
Thermogravimetric analysis (TGA) (**a**) and the derivative thermogravimetry (**b**) of the PTFE membrane, GOIM and CNIM-f.

The TGA and the derivative thermogravimetry curves of the PTFE membrane, GOIM and CNIM-f are shown in Figure 4a,b, respectively. At moderate temperatures (T < 150 °C), all membranes seemed relatively stable. The decomposition of PP resulted in the initial weight loss of the membranes at the temperature of ~350 °C [42], with significant weight change at the temperature of the 450–500 °C range. The significant weight loss was noticed again at a temperature of ~500 °C, and the major weight loss was seen at temperatures between 550 and 600 °C. This was due to the degradation of PTFE [43]. Overall, the TGA of modified membranes clearly shifted upward from the plain membrane, especially the CNIM-f. This may be implied that the modified membranes have a higher thermal stability than the unmodified ones. Higher thermal stability or the reduction of the thermal degradation of GOIM and CNIM-f could be attributed to the role of GO and CNTs that act as a radical scavenger and sacrificial agent, and their ability in inducing the barrier effect and high thermal conductivity [44,45]. However, between GOIM and CNIM-f, the latter had higher thermal stability. This is in accordance with the thermal stability of GO and CNTs, as reported in the previous publication [46].

**Figure 5:**
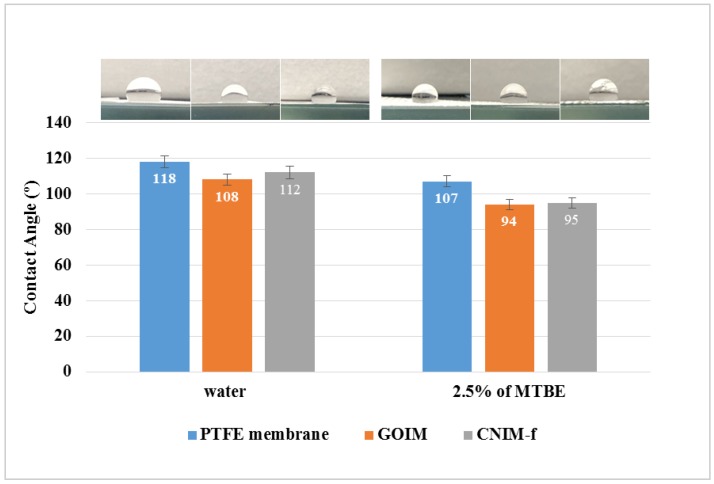
Photographs and contact angle of water and aqueous MTBE (2.5 wt %) drops on PTFE membrane, GOIM and CNIM-f.

The contact angles of pure water and aqueous MTBE solution on the membrane surface, along with their photos, are presented in Figure 5. The presence of GO and f-CNTs clearly resulted in the alteration of the contact angle of the drops on the PTFE membrane. Testing with deionized water, the contact angle for the plain membrane was 118°, which decreased to 108° and 112° for GOIM and CNIM-f, respectively. The reduction in the contact angle for GOIM and CNIM-f were attributed to the polar epoxy, carboxyl and hydroxyl functional groups on GO, and the carboxylic groups of f-CNTs. These enhanced the hydrophilicity of the membranes. This is in line with the previous publications [23,31,33]. For the MTBE solution, the contact angle for all membranes reduced as expected due to incorporation of the organic moiety in the aqueous solution, which decreased surface tension and eventually led to lower contact angles. For GOIM and CNIM-f, similar contact angle values of 94° and 95° were observed, and it was 107° for the plain membrane. Furthermore, the additional reduction of the contact angles for GOIM and CNIM-f compared to that for the plain membrane was attributed to the enhanced interaction of the organic moiety with GO and f-CNTs. Surface free energy of the original PTFE membrane and modified membranes was investigated using the contact angles of different liquids, as mentioned before. The surface free energy showed an increase with the membrane modification, using GO and f-CNTs. The surface free energy was 5.54 mJ/m^2^ for the plain membrane, and rose to 8.77 and 7.64 mJ/m^2^ for GOIM and CNIM-f, respectively. Although the surface energy of a particular material depends upon various factors, in general, the high surface free energy of modified membranes signify strong molecular attractions, while the low surface free energy of plain membrane indicates weaker attractive forces between the liquid and the membrane surface.

The measurement of liquid entry pressure (LEP) for plain and modified membranes was made using DI water and 2.5% of the MTBE solution as feed solutions. The LEP of the membranes tested with DI water were 67, 63 and 64 psig, and reduced to 37, 29 and 34 psig when MTBE was used as a feed for the plain membrane, GOIM and CNIM-f respectively. The reduction of LEP of the membranes when tested with MTBE was attributed to decrease in the surface tension of the liquid and the contact angle. This is due to the fact that LEP is proportional to the surface tension of the liquid and the degree of contact angle, as described in the literature [47]. The LEP of GOIM and CNIM-f were lower than that of the plain PTFE membrane, due to their lower contact angles due to the reasons given above.

### 3.1. SGMD Performance Using GOIM, CNIM-f and PTFE Membrane

In this study, MTBE permeate flux and MTBE removal efficiency were used to evaluate the performance of the modified membranes and compared to a plain PTFE membrane. The overall permeate flux of MTBE (*J_MTBE_*), across the membrane can be expressed as:

(1) $$J_{MTBE}=\frac{\left(W_{0}-W_{t}\right)}{A*t}$$

where, *W*_0_ and *W_t_* are the total mass of MTBE of the feed at the beginning of the experiment and after collection time *t*, respectively. *A* is the active membrane surface area. The MTBE removal efficiency can be measured according to the equation below.

(2) $$\%\;\mathrm{MTBE}\;\mathrm{removal}=\frac{\left(W_{0}-W_{t}\right)}{W_{0}}\times 100$$

**Figure 6:**
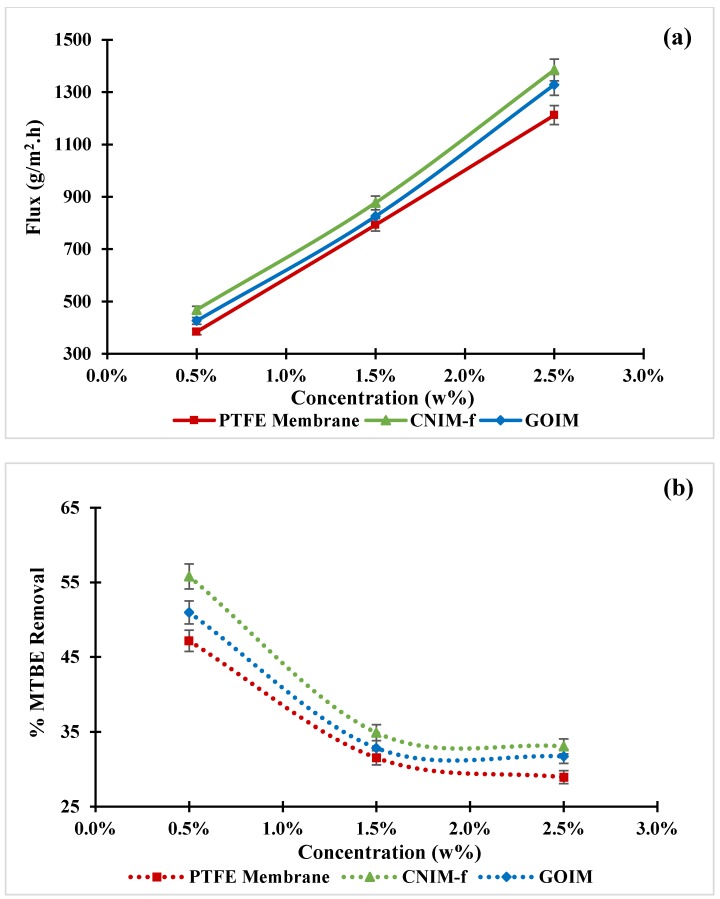
(**a**) Effect of feed concentration on MTBE flux, and (**b**) % MTBE removal as the function of feed concentration at the feed temperature of 30 °C and the feed flow rate of 20 mL/min.

The results of varying feed concentration on MTBE flux and percent MTBE removal are shown in Figure 6a,b, respectively. The MTBE feed concentrations of 0.5 wt %, 1.5 wt % and 2.5 wt %, with a constant feed flow rate of 20 mL/min and a temperature of 30 °C, were examined. From Figure 6a, it can be seen that the MTBE flux increased substantially with an increase in feed concentration for all membranes, and the flux of CNIM-f and GOIM were superior to that of the original membrane. The higher flux for CNIM-f and GOIM may be explained by the fact that f-CNTs and GO are the exceptional sorbents for the organic molecule [48], and lead to the improvement of the partition coefficient of MTBE. However, the high flux of CNIM-f over GOIM may be ascribed to the activated diffusion via adsorption and desorption on the CNT surface that facilitate faster MTBE transfer across the membrane [32,49]. The highest MTBE flux from CNIM-f was 1.4 kg/m^2^ h at the feed concentration of 2.5 wt %, while the maximum enhancement of 21.7% and 11% was obtained at the feed concentration of 0.5 wt %, compared to the PTFE membrane and GOIM, respectively. As shown in Figure 6b, the percent MTBE removal declined with the increment of the feed concentrations for all membranes. Both CNIM-f and GOIM still provided the higher percent removal than the plain membranes. The highest removal of 56% was observed for CNIM-f at the feed concentration of 0.5%.

**Figure 7:**
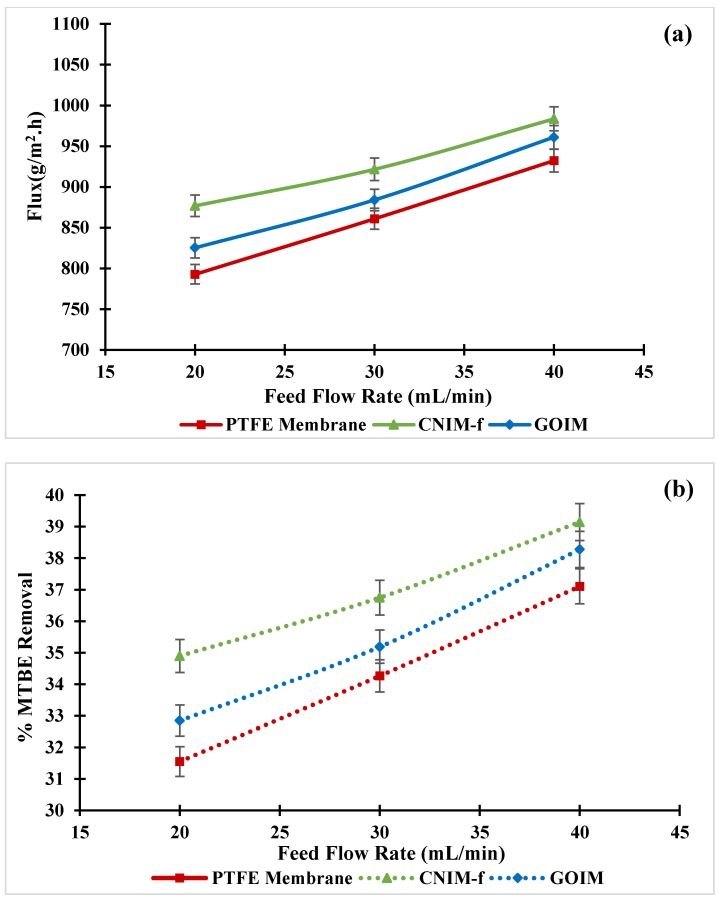
(**a**) Effect of feed flow rate on MTBE flux, and (**b**) % MTBE removal as the function of feed flow rate at the feed concentration of 1.5 wt % and the feed temperature of 30 °C.

The effects of the feed flow rate on MTBE flux and percent MTBE removal at 30 °C feed temperature and concentration of 1.5 wt % MTBE are shown in Figure 7a,b, respectively. The feed flow rate was varied between 20–40 mL/min. As can be seen in Figure 7a, it is clear that the MTBE flux steadily rose with an increase in the feed flow rate in all membranes. The increment of the feed flow rate increased the amount of MTBE and the availability of vapor into the MD module per unit time. The increased velocity also led to the reduction of temperature and concentration polarization, and consequently resulted in the permeate flux enhancement [50]. The modified membranes gave higher flux than the original membrane for all flow rates. The CNIM-f yielded the highest permeate flux of 0.98 kg/m^2^ h at the 40 mL/min feed flow rate, while the largest enhancements found at the feed flow rate of 20 mL/min, which were 11% and 4%, equated to the PTFE membrane and GOIM, respectively. Like the permeate flux, the percent MTBE removal increased with an increase in the feed flow rate as shown in Figure 7b. The CNIM-f presented the most percent removal, it was 39% at the same feed flow rate.

**Figure 8:**
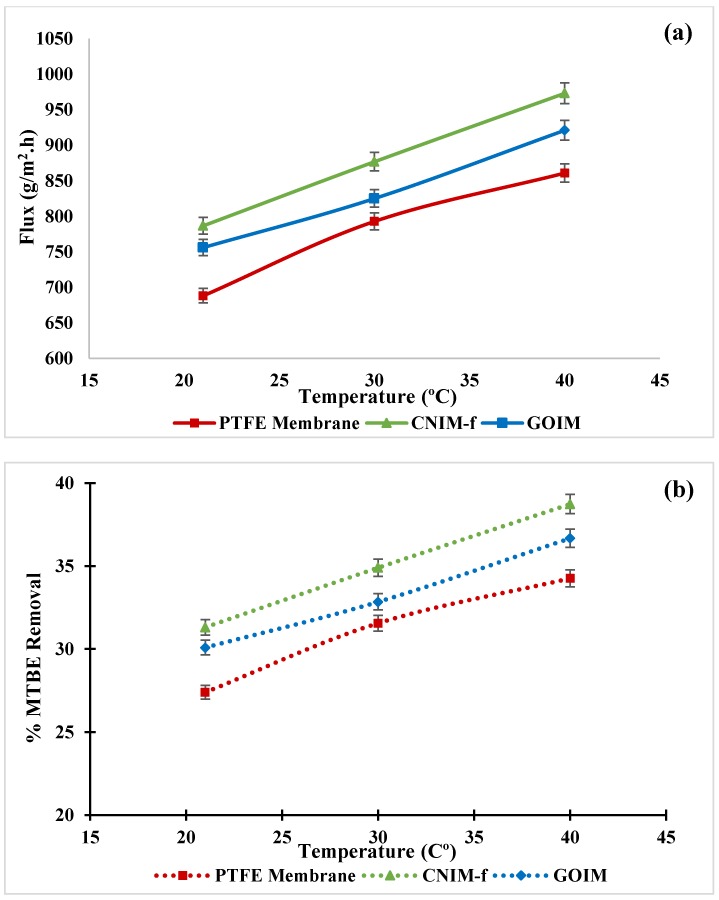
(**a**) Effect of feed temperature on MTBE flux, and (**b**) % MTBE removal as the function of feed temperature at the feed concentration of 1.5 wt % and the feed flow rate of 20 mL/min.

The results of varying feed temperature on MTBE flux and percent MTBE removal are presented in Figure 8a,b, respectively. The membrane performances were investigated at three different temperatures: 21, 30 and 40 °C at a constant feed flow rate of 20 mL/min, and at a feed concentration of 1.5%. As shown in Figure 8a, there is a clear trend of increasing the MTBE flux with an increase in temperature for all membranes. This is due to the fact that the vapor pressure rises with an increase in the temperature, according to Antoine’s equation, and then results in the extended vapor pressure difference between feed and permeate side, and consequently the enhancement in permeate flux [51]. Overall, the modified membranes showed better performance than that of the unmodified membrane for all temperatures. The highest flux of 0.97 kg/m^2^ h was obtained by CNIM-f at 40 °C, while the maximum enhancements found at 21 °C were 14% and 10%, over the PTFE membrane and GOIM, respectively. From Figure 8b, the percent MTBE removal also raised with the increment of temperature. CNIM-f reached the maximum efficiency with 39% removal at 40 °C, while GOIM and the pristine membrane were 37% and 34%, respectively.

The selectivity of MTBE separation can be expressed as:

(3) $$\mathrm{Selectivity}=\frac{y/\left(1-y\right)}{x/\left(1-x\right)}$$

where *x* and *y* are the weight fractions of MTBE in the feed and the permeate, respectively. 

**Figure 9:**
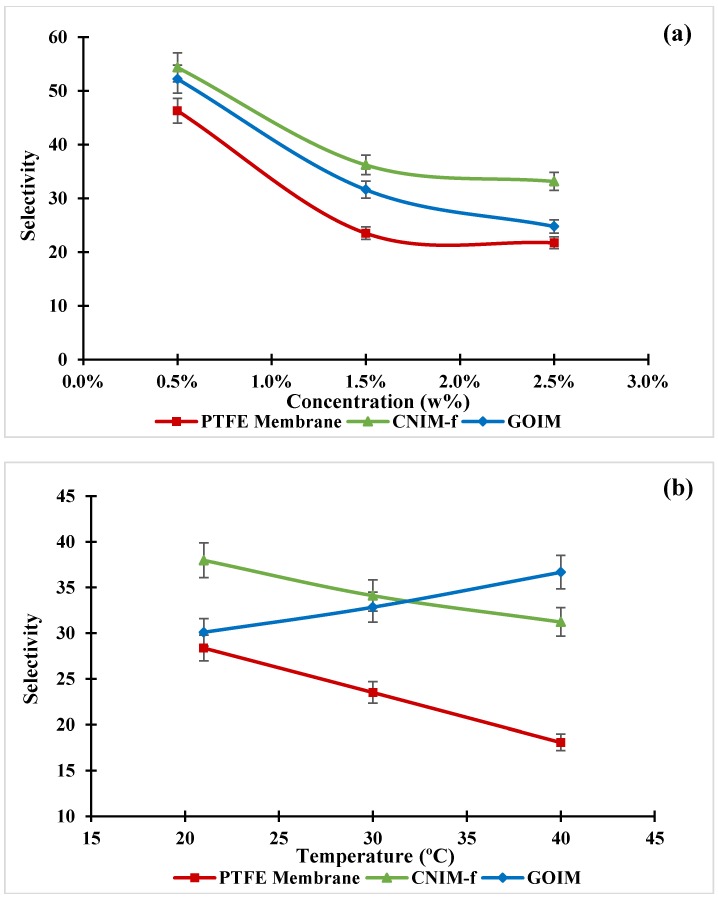
Effect of (**a**) feed concentration and (**b**) feed temperature on MTBE selectivity of membranes.

The effect of feed concentrations and temperatures on the MTBE selectivity of the membranes are presented in Figure 9a,b. In general, the MTBE selectivity of the modified membranes were superior to the original membrane for all operating parameters, and the MTBE selectivity of CNIM-f on average was higher than that of GOIM. The high selectivity of CNIM-f and GOIM over the original membrane is attributed to the facilitated transport of MTBE via f-CNTs and GO across the membrane that promote the membrane selectivity. As shown in Figure 9a, the selectivity of all membranes decreased with the increment of feed concentration. This was ascribed to the negative effect of viscosity [50]. The maximum selectivity of 54 was obtained for CNIM-f at the feed concentration of 0.5 wt %, while the average selectivity of CNIM-f, GOIM and the plain membrane were 41, 36 and 31, respectively. As can be seen from Figure 9b, the selectivity of the membrane, except GOIM, trended to reduce with an increase in the temperature. This may be due to the reduction in sorption capacity with temperature, which affects the overall selectivity. It is also possible that the negative effect of viscosity compensated for the effect of temperature increase, and lead to the selectivity decrease as mentioned in the articles [21,50]. The highest selectivity of 38 was also achieved by CNIM-f. Although the selectivity of GOIM had an opposite tendency to the others, its average selectivity did not have a significant difference from that of CNIM-f. The selectivity of GOIM was 33, whereas the selectivity of CNIM-f and the pristine membrane were 34 and 23, respectively. 

### 3.2. Mass Transfer Coefficients

The mass transfer coefficient (*k*) can be expressed as:

(4) $$k=\frac{J_{MTBE}}{\left(P_{f}-P_{p}\right)}$$

where *J_MTBE_* is the flux, *P_f_* and *P_p_* are the partial vapor pressures of the MTBE in the feed and permeate side, respectively. The partial pressure of MTBE on the feed side at a particular temperature was obtained from the calculation following Raoult’s Law. Since the permeate side of the membrane was dry air, the partial vapor pressure was considered as close to be zero. 

The permeate flux can be expressed by an Arrhenius-type equation, and the activation energy of the transport (*Ea*) can be calculated from this equation [52,53].

(5) $$J=A\;exp\;\left[-\frac{E_{a}}{RT}\right]$$

where *J* is permeate flux (mol m^−2^ h^−1^), *R* is the gas constant (J mol^−1^ K^−1^), and *T* is feed temperature (K).

Table 1 shows the variation of the mass transfer coefficients (*k*) of 1.5 wt % MTBE at various feed temperatures, and at a constant feed flow rate of 20 mL/min. With an increase in the feed temperatures, the k values of MTBE tended to decline. The CNIM-f offered the highest mass transfer coefficient, followed by GOIM and the plain PTFE membrane. The larger k values for CNIM-f was ascribed to the activated diffusion of MTBE on the f-CNT surface. The mass transfer coefficient enhancement of CNIM-f varied from 10% to 14%, compared to the plain membrane. The activation energy (*Ea*) for MTBE was calculated from the equation (5). The activation energies of PTFE membrane, CNIM-f and GOIM were 9, 8.6 and 7.9 kJ/mol, respectively. Although the Ea values for MTBE were lower than that has been reported for chloroform and butanol using MD [54,55], a direct comparison to those values may not be relevant due to the variations in the nature of chemicals, membranes and process parameters. At the same time, significantly lower values in the range of 1–3 kJ/mol have been reported for THF and methanol for pervaporative separation [56,57]. However, what is important is that the activation energy dropped with the CNT and GO modifications, indicating facilitated transport with these modifications. 

### 3.3. Membrane Stability

The thermal stability of the membranes was investigated by TGA as mentioned before. The results showed that there is no significant change happening with the membranes at the temperatures below 150 °C, which indicates the safe use of membranes at the operating temperatures of this study. The membrane wetting and change in the MTBE flux were also studied to evaluate the operational stability of the membranes for 30 days (8 hrs/day). No solvent leakage, as well as a change in MTBE flux, were observed over the period of time.

## 4. Proposed Mechanism

**Figure 10:**
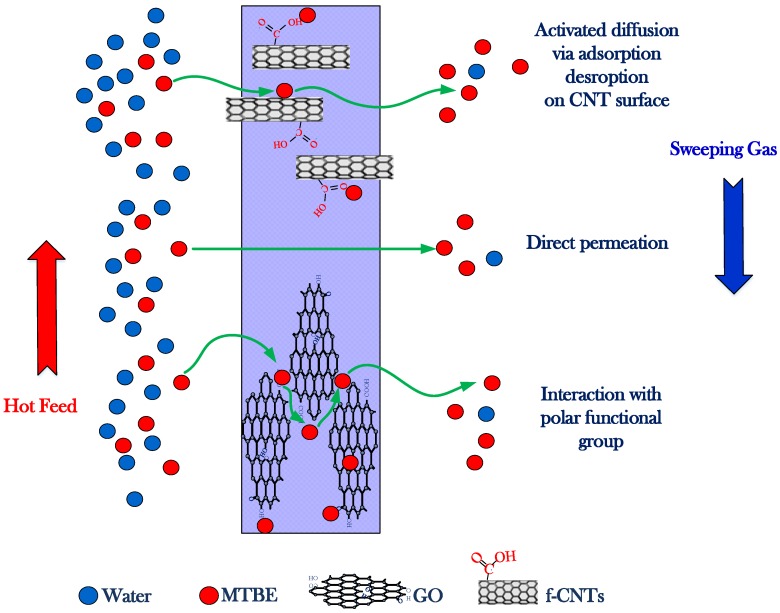
Schematic diagram for mechanism proposed on CNIM-f and GOIM.

The modified membranes, namely CNIM-f and GOIM, showed a significant enhancement in MTBE removal performance over the unmodified one. This could be attributed mainly to the fact that the f-CNTs and GO are excellent adsorbents for volatile organics [48]. The engineered nanocarbon provided high specific surface area, and served as sorption sites. In addition, the surface functional groups, such as the carboxylic group on f-CNTs, and epoxy, carboxyl, carbonyl and hydroxyl groups for GO, also play a major role as interaction sites for polar organic compounds like MTBE. Although it has been reported that, compared to CNTs, GO has a higher specific surface area [58], the CNIM-f provided the larger MTBE flux for this study. The formation of stacked graphene oxide layers on the membrane [59] could reduce the effective sorption sites and hinder the MTBE transport across GOIM, whereas the array of f-CNTs is expected to form as the pathway on CNIM-f, and fast adsorption-desorption on the CNT surface brings about the activated diffusion, and consequently enhances MTBE separation performances [60]. The proposed mechanism for MTBE flux enhancement by CNIM-f and GOIM is shown in Figure 10.

## 5. Conclusions

MTBE flux enhancement was achieved by using f-CNTs and GO-modified membranes. CNIM-f performance was superior to both GOIM and the original PTFE membrane in terms of flux, percent recovery, and mass transfer coefficients for all parameter variations. CNIM-f yielded the maximum flux of 1.4 kg/m^2^ h at the feed concentration of 2.5 wt %, and the highest percent enhancement and percent MTBE removal were 22% and 56% at the feed concentration of 0.5 wt %, respectively. Incorporation of nanomaterials also improved the membrane selectivity for MTBE, and the overall MTBE selectivity of CNIM-f was highest among the membranes. Immobilization of f-CNTs and GO on the plain membrane significantly enhanced partition coefficients, and that was confirmed by the reduction of the contact angle, which played a part in high MTBE transport. However, the higher MTBE flux for CNIM-f was attributed to the formation of f-CNTs array on the membrane and fast adsorption-desorption on the frictionless f-CNT surface that led to the activated diffusion.

## Figures and Tables

**Table 1:** Mass transfer coefficient of 1.5% MTBE feed solution at various feed temperatures with a feed flow rate of 20 mL/min.

Temperature (°C)	Mass Transfer Coefficient (kg/m^2^ s Pa) × 10^−6^		
	PTFE Membrane	GOIM	CNIM-f
21	2.19 ± 0.08	2.41 ± 0.08	2.51 ± 0.09
30	1.70 ± 0.02	1.77 ± 0.02	1.88 ± 0.03
40	1.29 ± 0.04	1.38 ± 0.04	1.45 ± 0.07
